# The impact of an inclusive education intervention on learning outcomes for girls with disabilities within a resource-poor setting

**DOI:** 10.4102/ajod.v9i0.555

**Published:** 2020-05-13

**Authors:** Mark Carew, Marcella Deluca, Nora Groce, Sammy Fwaga, Maria Kett

**Affiliations:** 1 Leonard Cheshire, London, United Kingdom; 2 UCL International Disability Research Centre, London, United Kingdom

**Keywords:** inclusive education, gender, disability, poverty, Kenya

## Abstract

**Background:**

Despite a global commitment to the right to education for persons with disabilities, little is known about how to achieve inclusive education in practice, particularly in low- and middle-income countries (LMICs), where the majority of the world’s people with disabilities reside. Moreover, although exclusion from education is magnified by intersecting gender and socioeconomic inequalities, there is especially little knowledge regarding what approaches to inclusive education are effective amongst girls with disabilities living in resource-poor settings.

**Objectives:**

The objective of this article was to assess the impact of an inclusive education intervention led by a non-governmental organisation (NGO) on the educational attainment of girls with disabilities in the resource-poor Lakes region of Kenya.

**Method:**

A quasi-experimental design was employed, where the literacy and numeracy educational attainment of the intervention and control groups was compared over two time points a year apart (Time 1 and Time 2; total matched *N* = 353). During this period, activities pertaining to six core components of a holistic inclusive education model were implemented.

**Results:**

Relative to the control group, girls with disabilities in the intervention group reported a greater increase in literacy and numeracy attainment, adjusted for grade and level of functional difficulty.

**Conclusion:**

Findings suggest that the intervention was successful in engendering additional improvements in the educational attainment of girls with disabilities from the resource-poor Lakes region of Kenya. Results highlight both the applicability of NGO-led interventions in settings, where national implementation of inclusive education is constrained, and the potential of taking such interventions to scale.

## Introduction

The United Nations (2007) Convention on the Rights of Persons with Disabilities has to date been ratified by 177 countries,^1^ signifying a global commitment to the rights of persons with disabilities, including the right to education (Article 24). Notwithstanding, in practice, gaps in the provision of education to children and adults with disabilities persist, with recent statistics suggesting that in some countries one in two children having a disability is not attending school regularly (UNESCO 2017). Moreover, the recent Sustainable Development Goals (SDGs) place emphasis on the provision of inclusive and quality education for all (SDG 4: ‘Ensure inclusive and quality education for all and promote lifelong learning’), and as such, along with other marginalised and excluded groups, the SDGs have the potential to change the landscape of people with disabilities in terms of their access to education (Department for International Development [DFID] 2000).

However, for low- and middle-income countries (LMICs), there are particular challenges in meeting these goals in practice. Conceptually, inclusive education originated in the Global North, and there is often much debate about how it should be implemented within many settings in LMICs (Miles & Singhal 2010). This leads to a disconnect between policy and practice. For example, Wapling (2016) notes that against the backdrop of relatively strong inclusive education policies in many settings (e.g. Cambodia, Southern Africa), in practice what is adopted is integration of children with disabilities into mainstream schools, with little attention to how other contextual realities, such gender and poverty, intersect with disability and impact access to education. Furthermore, the implementation of truly inclusive education models (i.e. one system for all children regardless of disability status) in LMICs may be constrained by a dearth of real resources, ineffective teacher training and absence of inclusive policies (Carew et al. 2018; Donohue & Bornman 2015; Kuyini & Desai 2007; Nkonyane & Hove 2014). For instance, where teachers are not provided with good quality training and equipment (e.g. teaching and learning aids) to help facilitate the inclusion of children with disabilities in mainstream classrooms, they may ultimately remain unwilling to adopt inclusion in practice (De Boer, Pijl & Minnaert 2011), despite agreeing with the goals and philosophy of inclusive education in the abstract.

Whilst there is a general need to understand, particularly in LMICs, what specific approaches work in terms of building blocks (i.e. teacher training; see, e.g., Bakhshi, Kett & Oliver 2013; Carew et al. 2018) that create an inclusive classroom (i.e. positive teacher attitudes to inclusion and adoption of inclusive teaching practices; see De Boer et al. 2011 for an example), ultimately, the ‘litmus test’ for identifying progress towards the goal of *inclusive* and *quality* education for all is if children with disabilities experience improvements in educational attainment whilst participating in inclusive classrooms, relative to their attainment in non-inclusive classrooms. This includes those children with disabilities who may experience more marginalisation relative to their peers. For instance, girls (as well as women) with disabilities are often described as possessing the ‘double disadvantage’ of experiencing marginalisation on the basis of both their gender and ability status (Fairchild 2002; Moodley & Graham 2015; Sheldon 2014). Moreover, poverty and deprivation are thought to magnify experienced inequalities, so that girls and women with disabilities living in LMICs, and, in particular, resource-poor areas in these settings, are likely to experience poorer outcomes relative to other groups (Emmett & Alant 2006). In the context of education, for instance, this is reflected in rates of education being lower amongst girls compared to boys with disabilities (UNESCO 2017). Girls with disabilities may also face specific and particularly distressing forms of marginalisation that impede their access to education such as sexual abuse in school (e.g. Caldas & Bensy 2014; Phasha & Nyokangi 2012) or forced marriage at an early age (Groce, Gazizova & Hassiotis 2014). Consequently, inclusive education models that are generally effective for children with disabilities may need additional components to allow the most marginalised of children with disabilities to access high-quality education. Often, this may necessitate a broader focus than just focusing on school and classroom. One example provided by Scior et al. (2016) is the role that parents and community members with intellectual disabilities may play in combating intellectual disability stigma, which is widespread compared to that encountered by other impairment groups. In a similar respect, inclusive education for girls with disabilities may necessitate engendering positive community attitudes about educational provision for both children with disabilities and girls in general.

In light of the current practical constraints in the implementation of inclusive education models in LMICs, there is a lack of empirical data on what specific approaches may provide children with disabilities with a quality education. In particular, less data are available on what enables learning for marginalised groups of children with disabilities in LMICs, including girls with disabilities from resource-poor regions of such countries. As delivery of inclusive education models in these areas is often fulfilled by non-governmental organisations (NGOs) (Carew et al. 2018), empirical analyses of such interventions can provide initial insight for governments and policy-makers into what approaches are effective in both real-life contexts and amongst the most marginalised of children with disabilities.

## Current research context

Although overall enrolment in primary education is increasing in Kenya because of the introduction of measures such as Free Primary Education in 2003 and increased social protection access, the number of girls with disabilities accessing primary education remains low, and the number of these girls dropping out of education is also increasing. The Lakes region of Kenya, located in the west, faces particular challenges in terms of deprivation, including in the context of education, and this deprivation disproportionally affects girls with disabilities. For example, between 2003 and 2009, the Lake region saw an increase of 12.5% in primary school enrolments (Kenya Ministry of Education 2017). However, in 2009, dropout rates rose to 9.2%, the highest in the country. Moreover, girls accounted for just 1.3% of all school attendants during this period, and although no representative data are available for the number of these girls who are disabled, it is assumed to be very small, given the extant work on disability and school attendance (e.g. Mizunoya, Mitra & Yamasaki 2018; UNESCO 2017). Moreover, even for the girls with disabilities who managed to attend and stay in school, existing research (e.g. Wapling 2016) has highlighted numerous sociocultural (e.g. attitudes) and school-level barriers that prevent such girls from staying away from schools and study and learn on an equitable basis with non-disabled peers. Disability is both a cause and a consequence of poverty (DFID 2000), meaning that the more impoverished Lakes region of Kenya likely contains a greater proportion of people living with disabilities compared to other regions of Kenya. For example, in two of its constituent sub-counties, Kisumu East and Mbita, it is estimated that approximately one-fifth to a quarter of girls aged 6–11 years have disabilities compared to a national average of 5% – 10% (Kenya National Bureau of Statistics 2005).

The demographics of the Lakes region of Kenya made it a suitable candidate for a field test of an inclusive education intervention developed by the UK-based NGO Leonard Cheshire. The research was funded by the UK government (Department for International Development) Girls Education Challenge (GEC) fund, designed to ‘…help up to a million of the world’s poorest girls improve their lives through education and find better ways of getting girls in school and ensuring they receive a quality of education to transform their future’ (https://www.gov.uk/guidance/girls-education-challenge).

Results from a dedicated training component designed to address teacher beliefs, attitudes and practices around inclusive education have already been reported in Carew et al. (2018).

The objective of this research was to assess the impact of the intervention on the educational attainment of girls with disabilities in the Lakes region of Kenya.

## Method

### Design

At the outset of the study, a scoping exercise was conducted to discern the barriers that girls with disabilities face to accessing education. Specific barriers identified by the scoping exercise included inaccessible school buildings; learning materials; teaching methods; and negative attitudes from parents, community members and teachers about disability in education. It was also found that disabled girls and their families did not receive the full necessary educational and rehabilitative support they needed to access mainstream education (e.g. help with additional costs). These findings were largely consistent with the extant literature on barriers to education that disabled children face within other resource-poor contexts (e.g. Wapling 2016). As a result of this scoping exercise, intervention activities were explicitly aligned with the identified barriers as part of a wider project theory of change.

**FIGURE 1:**
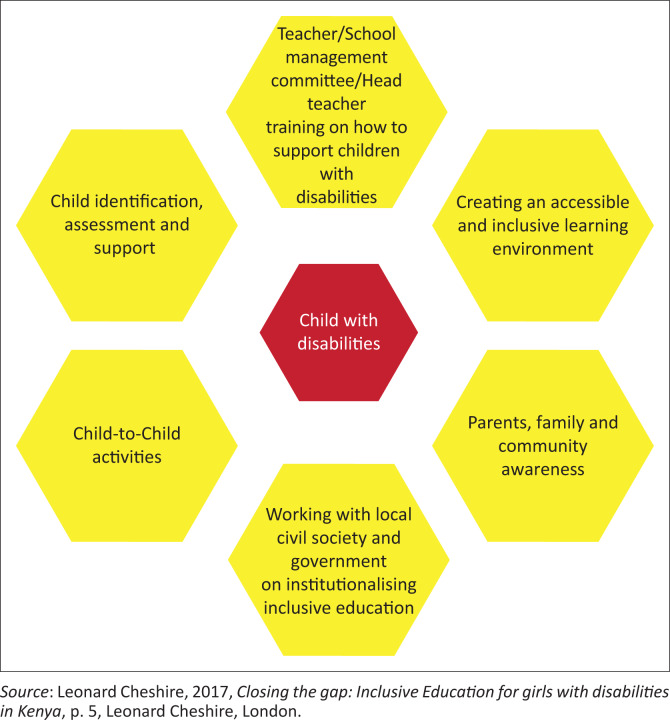
The (Organisation) inclusive education model.

The intervention implementer (Leonard Cheshire) is a UK-based global disability-focused NGO that supports disabled people’s access to education (as well as work and employment) in several countries around the globe, including in Southern and East Africa, where the organisation also has regional offices. The organisation’s intervention is based on a set of six main interlinked components (Leonard Cheshire 2017). Figure 1 displays the conceptual model. We summarise each of these components below in turn and provide examples of the activities undertaken under each component.

The first component was the creation of an accessible learning environment. This included building of ramps, widening of windows and fitting of translucent sheets to allow more light in classrooms, thus enabling those with low vision to see better, as well as providing assistive devices (e.g. wheelchairs and hearing aids) and teaching and learning materials.

The second set of activities concentrated on raising awareness about both disability and gender issues amongst caregivers and the community to challenge deeply rooted stereotypes and practices about disabled people in general and girls in particular (e.g. that they cannot learn or learn as capably as others). Specifically, a small group of purposively selected community members were trained on disability rights, gender issues and inclusive education, and subsequently cascaded this training throughout each community.

The third component of the project was the development and running of on-going child-to-child activities (i.e. peer support and after-school clubs) designed to promote integration and socialisation between girls with and without disabilities. The fourth component of the project trained teachers at project schools on inclusive education practices and disability rights (see Carew et al. 2018). The fifth component of the project supported the identification and assessment of disabled children (e.g. by liaising with external staff regarding unidentified children living with a disability). Finally, to ensure intervention benefits continued beyond the period of direct activity, the sixth component of the project advocated for policy change at the county and national levels (e.g. pushing for a review of the country-wide Special Needs Education policy).

To investigate the efficacy of the intervention on the educational attainment of girls with disabilities, this study adopted a quasi-experimental design, in which the impact of the intervention was assessed over two time points (Time 1 and Time 2). Specifically, a group of girls with disabilities who received the intervention were compared with a control group of girls with disabilities who did not receive the intervention.

### Participants

The Time 1 intervention sample comprised 406 girls with disabilities who were attending primary schools in the Lakes region of Kenya where intervention activities were being conducted. The primary school classes these girls were drawn from ranged from Grade 1 to Grade 8 (aged from 6 to 14 years) and the distribution by grade ranged from 8% to 17% of the sample. The most frequent disability reported was hearing impairment (*N* = 97; 24%), followed by visual impairment (*N* = 92; 23%). The Time 2 intervention sample comprised 289 girls (an attrition rate of 29%).

The Time 1 control sample comprised 108 girls with disabilities attending primary schools within the Lakes region of Kenya where intervention activities were not being conducted. These girls were drawn from the same range of primary school classes (i.e. Grade 1 to Grade 8) and each class contained 6% to 19% of the sample. The most frequent disability reported was physical disability (*N* = 37; 34%), followed by intellectual disability (*N* = 20; 19%). The Time 2 control sample comprised 64 girls (an attrition rate of 41%).

The smaller size of the control group is indicative of the substantive real-life barriers that girls with disabilities face in obtaining education in Kenya. That is, given that girls with disabilities are more likely than non-disabled peers to not attend school regularly in the absence of any intervention (UNESCO 2017), it was not possible to obtain a larger sample of control girls that could be meaningfully compared with the intervention group.^2^

### Measures

*Uwezo English literacy, Kiswahili literacy and numeracy test scores*: The *Uwezo* test is a pretested and validated tool administered within households across East Africa to assess learning in English and Kiswahili^3^ literacy as well as numeracy skills at lower primary levels (Uwezo 2009). In each of the three domains, the administered test contains several exercises that are given to participating children by trained assessors. Based on the competencies displayed by each child (i.e. in English literacy, Kiswahili literacy and numeracy), the assessor awards them a score. In our study, following *Uwezo* guidelines, the English and Kiswahili tests comprised five possible levels corresponding to the assessed competencies of the child. (‘nothing’, ‘letter’, ‘word’, ‘paragraph’ and ‘story’). The first four of these signify whether the child could read the descriptor in questions (e.g. letters), whilst the last signifies that the child could broadly comprehend the meaning of a passage of text. Similarly, following *Uwezo* guidelines, competency in numeracy was assessed using seven possible levels (‘nothing’, ‘counting [dots]’, ‘number recognition’, ‘addition’, ‘subtraction’, ‘multiplication’ and ‘division’).^4^ Additionally, all administered tests were converted to Braille to ensure that materials were accessible to girls with visual disabilities.

*Severity of disability*: The Washington Group short set of questions was used to measure the severity of disability (Madans, Loeb & Altman 2011). The questions are measured on a 4-point scale (1 = *No, No difficulty*, 4 = *cannot do at all*), and are asked whether respondents can complete a range of activities. Specifically, the following items were used: ‘Do you have difficulty seeing, even if wearing glasses?’; ‘Do you have difficulty hearing, even if using a hearing aid?’; ‘Do you have difficulty walking or climbing steps?’; ‘Do you have difficulty remembering or concentrating?’; ‘Do you have difficulty with self-care, such as washing all over or dressing?’

### Procedure

At its inception, the project firstly trained community resource workers, who collected data on primary enrolment in five sub-counties of the Lakes region (Kisumu East, Kuria East, Mbita, Migori and Siaya) and worked with Kenyan government-mandated Education Assessment Resource Centres (EARCs) to identify 2 500 girls with disabilities. The project also liaised with the Kenyan Government to select 75 schools (50 intervention and 25 control) across five counties, half of which would receive the intervention and the other half which would not receive the intervention.

The project employed an external evaluator who developed a sampling framework to ensure sample representativeness in each group (i.e. intervention and control) across counties and grades (i.e. class) and collected data from this subsample of girls. At both data collection points, quantitative data collection, including *Uwezo* assessment, was carried out at the girls’ households (with consent from their caregiver) by specially trained data collectors. Time 1 data were collected at the end of 2015 (i.e. November–December) over a period of a month, whilst Time 2 data were collected a year later at the end of 2016 (i.e. November–December), also over a month period.

At the conclusion of the project in early 2017, data were provided to the authors who conducted further secondary analysis of the project’s midline and endline data (see below).^5^

### Analytical strategy

We used a difference-in-difference approach to assess the impact of the intervention over the studied period by comparing the difference in *change* (i.e. Time 2 – Time 1) in the girls’ respective learning score (i.e. English, Kiswahili and numeracy) within the intervention and control groups. Our analyses were performed using the statistical package SPSS version 24.

The choice of analytic strategy was influenced by natural limitations present in our data in light of its field-based settings related to non-random differences between the groups. Specifically, although data were collected from a pre-intervention baseline group of girls with disabilities, over three-quarters were subsequently assigned by the Kenyan system to schools outside the intervention areas and had to be substituted at the subsequent data collection points. Consequently, this study reports on findings from a sample of girls with disabilities only from the midline (i.e. Time 1) and the endline (Time 2) project phases. As such, the intervention group had already been exposed to some of the intervention activities at the first assessment point, although intervention activities continued through the project duration. Accordingly, at Time 1, analysis (controlling for grade) revealed that English and Kiswahili scores were higher in the intervention group relative to the control group (range: *p* = 0.003–0.046), which is consistent with a potential positive impact of the intervention prior to Time 1. There was no differences in numeracy scores between the groups (*p* = 0.192).

Secondly, initial analysis revealed that at Time 1, the intervention and control samples differed in their grade compositions, with those in the intervention group belonging to a significantly higher grade compared to the control (*p* = 0.018).

Finally, the intervention and control groups differed in the functional difficulty caused by their disability. That is, the intervention group reported significantly more difficulty with both seeing and hearing relative to the control, whilst for difficulty in walking, the reverse was identified (all *p* < 0.001). There was no significant difference between the difficulty in concentrating and difficulty with self-care (range: *p* = 0.270–0.757). This was likely as there were more girls with an assessment of visual and hearing impairment in the intervention group (24% and 23% of the sample, respectively) compared to the control (14% and 12%), whilst girls with physical disabilities were underrepresented (13% in the intervention group vs. 34% in the control group). This is also likely because of the substitution of cases from baseline described above (i.e. the project had to select girls that were enrolled within intervention schools).

The application of the difference-in-difference methodology allows for the evaluation of interventions even where there is extant cross-group selection bias (i.e. differing characteristics), as in this case (Gertler et al. 2016). That is, instead of comparing post-intervention outcomes between intervention and control groups, which may be influenced by previously existing outcomes (measured and unmeasured variation), the difference-in-difference approach compares the *change* in outcomes over time in the intervention group with the comparison control group. Thus, a key assumption is that the comparison group must accurately represent change in outcomes that would have occurred in the absence of any intervention, not that there is equivalence between the groups at the outset of measurement (Gertler et al. 2016).

### Reflexivity

In terms of Global North–Global South collaboration, partnership has been problematised, particularly in terms of its model of capacity building, which often implicitly denotes Global South actors as the beneficiaries of interventions and the Global North as providers and thus creates a power asymmetry (Binka 2005). With this in mind, two broad points are relevant to be raised about the intervention and our analyses.

The first point is that although project funding and implementing NGO stem from the United Kingdom, the intervention activities were designed and facilitated by Kenyan team members based locally at a regional office in Western Kenya, with input from UK-based colleagues. Similarly, the external evaluators of the project were Kenyan. Within disability inclusive development and development more broadly, we (i.e. the authors) view the equitable involvement of actors based within contexts and with experiences of the identities (e.g. disability) under study as crucial to the meaningful implementation and assessment of interventions, although there is a natural debate about what equitable participation would constitute to different actors.

In relation, the second point we wish to highlight is our (i.e. the authors) identities. We are a group of three women and two men, and one of us identifies as having a physical disability. One of us is Kenyan and worked on the intervention implementation and assessment in the Lakes region, whilst the remaining authors are academics from the Global North (the UK, the USA and Italy) who work at a research centre formed through a partnership between the implementing NGO and a university. Three of us have over 20 years’ experience of disability inclusive development, whilst the remaining two are more early-career. We anticipate (and indeed optimistic) that these mix of identities mean that we have contributed useful perspectives to the key debates raised by our findings, but this is obviously up to individual readers to decide.

### Ethical consideration

Ethical approval to conduct secondary data analysis of the data collected throughout the project was granted by University College London (Ethical clearance No.: ID: 1661/005). Additionally, at both Time 1 and Time 2, the external evaluator provided a declaration to the NGO (Leonard Cheshire) that the data were collected in an ethical manner, following the protocols set out by the funder.

## Results

Table 1 shows the mean values (M) and standard deviation (SD) of *Uwezo* test scores. Findings are presented in two sections. Firstly, we check for the impact of panel attrition on our sample. Secondly, addressing our main objective, we assess the unique contribution of the inclusive education intervention to the *Uwezo* scores of girls using longitudinal regression models.

**TABLE 1:** Mean values and standard deviations of *Uwezo* test scores.

Measure	Pre-intervention		Post-intervention	
	Mean	SD	Mean	SD
**Intervention**				
English literacy	3.63	1.41	3.80	1.27
Kiswahili literacy	3.53	1.54	3.84	1.40
Numeracy	4.12	1.30	4.38	1.15
**Control**				
English literacy	3.17	1.48	3.22	1.34
Kiswahili literacy	2.88	1.67	3.11	1.47
Numeracy	3.79	1.45	4.00	1.45

### Panel attrition

Differences between the participants who responded at Time 2 and the full Time 1 data set were checked separately for each group. For the intervention group, differences were non-significant across all key measures (range: *p* = 0.061–0.777), bar difficulty in seeing. Respondents had more difficulty in seeing (*M* = 1.72, SD = 0.81) compared to non-respondents (*M* = 1.50, SD = 0.71), *F* (1, 404) = 6.98, *p* = 0.009, partial *η*^2^ = 0.017.

For the control group, attrition produced three significant differences on key measures. Firstly, respondents had less difficulty in remembering or concentrating (*M* = 1.66, SD = 0.89) compared with non-respondents (*M* = 2.02, SD = 1.00), *F* (1, 106) = 3.97, *p* = 0.049, partial *η*^2^ = 0.036, and less difficulty with self-care (*M* = 1.16, SD = 0.37) compared with non-respondents (*M* = 1.41, SD = 0.66), *F* (1, 106) = 6.53, *p* = 0.012, partial *η*^2^ = 0.058. Finally, respondents had significantly higher English *Uwezo* scores (*M* = 3.41, SD = 1.34), compared with non-respondents (*M* = 2.82, SD = 1.60), *F* (1, 106) = 4.27, *p* = 0.041, partial *η*^2^ = 0.039.

As the majority of non-respondents at Time 2 were girls who had dropped out of school, these differences were not surprising. That is, girls experiencing more functional difficulty (i.e. disability) than their peers were often at more risk of dropping out of school (Mizunoya et al. 2018), hence the need to conduct interventions.

### Impact of the inclusive education intervention on learning scores

To assess the impact of the inclusive education intervention on the learning scores of girls with disabilities, we ran three longitudinal regression models. Each model regressed the *change* in a learning score over time (i.e. Time 2 – Time 1 English, Kiswahili or numeracy) onto groups (intervention and control). Additionally, we also controlled for the influence of grade and the level of functional difficulty the girls experienced across each Washington group domain (seeing, hearing, walking, remembering and self-care).

The model regressing the change in English learning scores on the predictors explained a small amount of variance (*R*^2^= 0.06), *F* (7, 345) = 3.31, *p* = 0.002. As hypothesised, there was a significant and positive association between group and the change in English learning scores over time (*B* = 0.49, *β* = 0.17, *t* = 2.95, *p* = 0.003). That is, compared with the control group, the intervention group experienced a greater increase in English scores. The only other significant predictor of the change in English learning scores was grade (*B* = -0.10, *β* = -0.19, *t* = -3.63, *p* < 0.001). Specifically, the higher the grade of the participant, the less their English learning score changed over time. No other predictors were significant in the model (range: *p* = 0.109–0.891).

The model regressing the change in Kiswahili learning scores on the predictors explained a small amount of variance (*R*^2^ = 0.08), *F* (7, 345) = 4.54, *p* < 0.001. As predicted, there was a significant and positive association between group and the change in Kiswahili learning scores over time (*B* = 0.41, *β* = 0.12, *t* = 2.20, *p* = 0.029). In other words, compared with the control group, the intervention group experienced a greater increase in Kiswahili scores. The only other significant predictor of the change in Kiswahili learning scores was grade (*B* = -0.14, *β* = -0.24, *t* = -4.55, *p* < 0.001). Specifically, the higher the grade of the participant, the less their Kiswahili learning score changed over time. No other predictors were significant in the model (range: *p* = 0.150–0.477).

The model regressing the change in numeracy learning scores on the predictors explained a small amount of variance (*R*^2^ = 0.06), *F* (7, 345) = 3.30, *p* = 0.002. As hypothesised, there was a significant and positive association between group and the change in numeracy learning scores over time (*B* = 0.48, *β* = 0.14, *t* = 2.40, *p* = 0.017). Namely, compared with the control group, the intervention group experienced a greater increase in numeracy scores. There were two other significant predictors of the change in numeracy learning scores, grade (*B* = -0.09, *β* = -0.15, *t* = -2.74, *p* = 0.007) and difficulty in walking (*B* = 0.34, *β* = 0.16, *t* =2.78, *p* = 0.006). Specifically, the higher the grade of participants, the less their numeracy learning score changed over time, whilst the more the difficulty participants had in walking, the more their numeracy learning score changed over time. No other predictors were significant in the model (range: *p* = 0.126–0.846).

## Discussion

Our findings reveal that, over the intervention period, girls with disabilities who participated in the inclusive education intervention obtained significantly higher English, Kiswahili and numeracy test scores compared with a comparable group of girls with disabilities who did not participate in the intervention. Moreover, the longitudinal association between group and *Uwezo* test scores was present when controlling for severity of disability and primary school grade. This suggests that the intervention was effective for both girls who experienced different degrees of impairment and girls who were at different stages of primary school learning.

The evidence generated by this study suggests that a holistic inclusive education model (Organisation 2017), encompassing intervention activities within both schools and wider community, could benefit the learning outcomes of children with disabilities. It is also encouraging that the intervention was effective when tested in the field among girls with disabilities from a resource-poor region of a LMIC, as the literature has identified that both gender and poverty intersects with disability to create additional barriers in multiple areas of social participation, including education (e.g. Emmett & Alant 2006).

Findings from inclusive education interventions delivered by NGOs, particularly when theory-led, are a useful step to explore ‘what works’ in practice, especially given that the implementation of inclusive education interventions by other actors could be constrained by a lack of resources (e.g. Donohue & Bornman 2015; Kuyini & Desai 2007). From this study, two observations for the future research are relevant in this respect. Firstly, the intervention conducted by (Organisation) was holistic, containing six interlinked core components comprising a range of related activities (see Organisation 2017). The future research should also examine the unique impact of each component (e.g. comparing the impact of hours of input per component on learning outcomes) to clarify what contributes the most to improvement in learning outcomes and thus what could be prioritised in situations where resources to implement full inclusive education models are unavailable. Secondly, it is worth highlighting that despite the scale and range of activities conducted as part of the intervention, the intervention explained only 6% – 8% of the variability in learning outcomes. This highlights the difficulty of achieving inclusive education in practice, where educational attainment could be influenced by a range of factors that were not measured in this study. Thus, the future research should continue to explore and test additional determinants of learning scores, although many are likely to be sociocultural and specific to the context under study (i.e. the Lakes region).

In conducting this study, we encountered some challenges that arose because of its field settings. Specifically, it was initially planned to test the impact of the intervention over three time points, but after the baseline many of the girls were subsequently allocated to schools outside project areas, requiring re-sampling at the midline project phase (i.e. Time 1). Girls were not therefore allocated randomly to the intervention and control groups, and consequently, there was cross-group variation in their level of functional difficulty and grade composition, although these were controlled for in the analyses. Similarly, as girls in the intervention group were already exposed to activities prior to Time 1, the analyses reflect the impact of intervention over the assessed period (i.e. Time 1 to Time 2), not its overall impact on girls with disabilities.

## Conclusion

The findings shed light on the effectiveness of a holistic inclusive education intervention conducted in the field amongst a marginalised group of children with disabilities in Kenya (i.e. girls with disabilities from the resource-poor Lakes region). Results suggest that the intervention engendered additional improvements in the learning outcomes of marginalised children with disabilities, providing both a promising avenue for government-led scale up in the Lakes region and highlighting the application of NGO-led interventions to build evidence in settings where national implementation of true inclusive education models is constrained (e.g. by lack of resources). Future research is needed to discern what elements of inclusive education implementation should be prioritised in such contexts. In this respect, we anticipate that our findings are helpful in stimulating further work in this area.

## Footnotes

1 https://www.un.org/development/desa/disabilities/convention-on-the-rights-of-persons-with-disabilities.html.

2 The control group sample reported in this article comprises part of a larger control sample not receiving the intervention which included responses from out of school girls. However, as these girls do not attend school, they do not constitute a meaningful comparison group (i.e. girls attending school but not receiving the intervention).

3 Although not the main language spoken in the Lakes region, which is Luo, Kiswahili is a national language and the main language of instruction within schools, along with English

4 At Time 1, only the first five levels of numeracy were assessed. However, we obtained ceiling effects, that is, almost two-thirds (57%) of the entire surveyed cohort were judged to have competency in subtraction, two additional levels were added at Time 2.

5 Summary statistics of the external evaluation are displayed at: https://girlseducationchallenge.org/. They show that the intervention achieved 165% of its literacy target and 171% of its numeracy target over the same period as detailed in this article (i.e. midline to endline).
